# Supplementary figures and images for: The bright side of pessimism: Promoting wealth redistribution under (felt) economic hardship
Source: PLoS One. 2020 Dec 14;15(12):e0243486. doi: 10.1371/journal.pone.0243486 (PMC7735630; doi:10.1371/journal.pone.0243486)

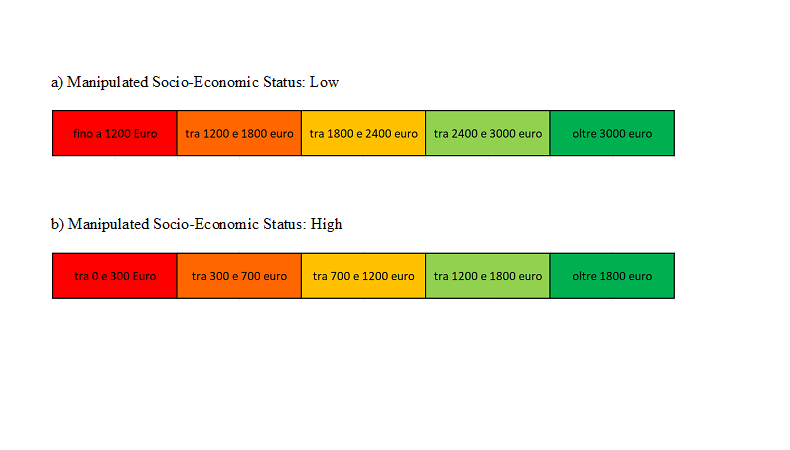

Supplement: S1 Fig — (TIF) [file pone.0243486.s001.tif]
