# Supplementary material for: The bright side of pessimism: Promoting wealth redistribution under (felt) economic hardship
Source: PLoS One. 2020 Dec 14;15(12):e0243486. doi: 10.1371/journal.pone.0243486 (PMC7735630; doi:10.1371/journal.pone.0243486)
Supplement: S1 File — Scale used to assess participants’ expected risks. (PDF) [file pone.0243486.s002.pdf]

a) Personal Mind-Set condition

At some point in their life, all people, are exposed to potential risks and benefits, which may depend on genetic traits, family history, lifestyle, life habits, or other factors.

You will find a list of positive and negative events. Please, indicate, for each event, the probability that it may happen to you personally in the next twenty years, compared to the average of persons of your same age and sex.

Compared to the average of persons of your same age and sex, what is the probability that in the next twenty years you……

	-3 absolutely below average			0 within the average			+3 absolutely above average
getting divorced (G)	○	○	○	○	○	○	○
successfully managing economic problems (E) R	○	○	○	○	○	○	○
being in a car accident (G)	○	○	○	○	○	○	○
asking for a loan to pay off a debt (E)	○	○	○	○	○	○	○
developing drinking problems (G)	○	○	○	○	○	○	○
getting a very good salary (E) R	○	○	○	○	○	○	○
job loss or getting fired (E)	○	○	○	○	○	○	○

(G) = general risk; (E) = economic risk; R = reverse coded

b) Collective Mind-Set condition

At some point, all nations are exposed to potential risks and benefits, which may depend on geographical features, history, internal political affairs, international economic affairs, or other factors.

You will find a list of positive and negative events. Please, indicate, for each event, the probability that it may happen to Italy in the next twenty years, compared to the average of OCSE nations (i.e., the most developed countries). NOTE: in the ranking of the OCSE countries, Italy is currently around the middle.

Compared to the average of OCSE nations, what is the probability that in the next twenty years in Italy……

	-3 absolutely below average			0 within the average			+3 absolutely above average
occurring a grave flood (G)	○	○	○	○	○	○	○
number of families with economic problems will be… (E)	○	○	○	○	○	○	○
occurring a powerful earthquake (G)	○	○	○	○	○	○	○
number of families asking for a loan to pay off a debt will be… (E)	○	○	○	○	○	○	○
solving problems of waste (G) R	○	○	○	○	○	○	○
number of families relying on a very good salary will be… (E) R	○	○	○	○	○	○	○
number of families without income because of job loss or dismissal will be… (E)	○	○	○	○	○	○	○

(G) = general risk; (E) = economic risk; R = reverse coded
